# Consolidation deficits in episodic memory define distinct clinical and neurodegenerative profiles in Huntington’s disease

**DOI:** 10.1016/j.nicl.2025.103894

**Published:** 2025-10-28

**Authors:** Saul Martinez-Horta, Angela Quevedo-García, Arnau Puig-Davi, Frederic Sampedro, Javier Oltra-Cucarella, Jesús Pérez-Pérez, Carla Franch-Martí, Gonzalo Olmedo-Saura, Elisa Rivas-Asensio, Anna Vazquez-Oliver, Laura Pérez-Carasol, Andrea Horta-Barba, Javier Pagonabarraga, Jaime Kulisevsky

**Affiliations:** a Movement Disorders Unit, Neurology Department, Hospital de la Santa Creu i Sant Pau, Barcelona, Spain; b Biomedical Research Institute Sant Pau (IIB-Sant Pau), Barcelona, Spain; c Centro de Investigación Biomédica en Red-Enfermedades Neurodegenerativas (CIBERNED), Spain; d Department of Medicine. Autonomous, University of Barcelona, Spain; e European Huntington’s Disease Network (EHDN), Spain; f Neuroradiology Department, Hospital Vall d'Hebron - Institut de Diagnostic per la Imatge, Barcelona, Spain; g Departamento de Psicología de la Salud, Universidad Miguel Hernández de Elche, Elche, Spain

**Keywords:** Huntington’s disease, Neuropsychology, Episodic memory, Hippocampus, MRI

## Abstract

**Background:**

Huntington’s disease (HD) is primarily associated with executive dysfunction, but episodic memory impairment is also present. Traditionally, these memory deficits have been attributed to retrieval difficulties linked to fronto-striatal dysfunction, rather than to disruptions in encoding or consolidation processes. However, the specific nature and diversity of memory impairments in HD remain underexplored.

**Objective:**

To characterize the profile of episodic memory impairment in HD, identify distinct cognitive phenotypes, and examine their clinical, neuroanatomical, and biomarker correlates.

**Methods:**

We assessed episodic memory in HD patients and healthy controls using the Free and Cued Selective Reminding Test (FCSRT), complemented by Item-Specific Deficit Approach (ISDA) indices to quantify encoding, consolidation, and retrieval deficits. Structural MRI was used to identify gray matter volume correlates, and plasma neurofilament light chain (NfL) was measured as a marker of neuroaxonal injury.

**Results:**

Compared to controls, HD patients showed marked impairments in free recall with preserved cued recall, suggesting predominant retrieval deficits. However, nearly one-third of patients exhibited global impairments across all FCSRT components, mainly driven by consolidation deficits consistent with medial temporal lobe dysfunction. This subgroup also showed worse cognitive and functional performance and significant atrophy in the hippocampus, entorhinal cortex, and parahippocampal gyrus.

**Conclusion:**

Episodic memory dysfunction in HD is heterogeneous and includes both retrieval-related and consolidation-driven profiles. These profiles reflect distinct neurodegenerative patterns, emphasizing the importance of cognitive subtyping for improving clinical characterization and biomarker development in HD.

## Highlights

• Distinct memory profiles in HD reflect divergent trajectories of brain atrophy.
• Retrieval deficits predominate but a subgroup shows marked consolidation failure.
• Medial temporal atrophy underlies severe consolidation deficits.
• Plasma NfL fails to reflect regional memory-related brain degeneration.
• Neuroimaging uncovers non-frontostriatal trajectories in HD cognitive impairment.

## 1 Introduction

Huntington’s disease (HD) is an autosomal dominant, monogenic neurodegenerative disorder caused by the expansion of CAG repeats in exon 1 of the HTT gene on chromosome 4. This mutation triggers a complex cascade of pathological processes, leading to progressive neurodegeneration primarily affecting the basal ganglia, cerebral cortex, and white matter tracts throughout disease progression (Gregory et al., 2020, Johnson et al., 2021, Rosas et al., 2008, Tabrizi et al., 2011). These brain changes underpin the emergence of the hallmark symptoms of HD in the form of motor abnormalities, cognitive decline, and behavioral disturbances (Ross et al., 2014)

While cognitive and behavioral alterations in HD have classically been described as reflecting a prototypical prefrontal syndrome stemming from the disruption of frontostriatal circuits due to striatal atrophy, growing evidence indicates a broader cognitive phenotype (Martinez-Horta et al., 2016, Martinez-Horta et al., 2020, Snowden, 2017). Neuropathological and neuroimaging studies have demonstrated that degeneration in HD extends beyond the basal ganglia, involving temporoparietal and occipital cortices (Martinez-Horta et al., 2020). In parallel, cognitive studies reveal that many patients exhibit impairments beyond executive functions, affecting visuospatial processing, language, and episodic memory (Glikmann-Johnston et al., 2019, Lawrence et al., 2000, Martinez-Horta et al., 2023, Say et al., 2011). Together, these findings challenge the notion that cognitive dysfunction in HD is restricted to a frontal-subcortical model and suggest that multiple cognitive systems may be implicated. Importantly, broader cognitive involvement in HD has been associated with a worse prognosis, reflecting more widespread brain degeneration and potentially different coexisting pathological mechanisms, including tau-related pathology (Martinez-Horta et al., 2024, Martinez-Horta et al., 2020, Ruiz-Barrio et al., 2024, Vuono et al., 2015). However, despite these observations, the characterization of cognitive phenotypes of HD beyond executive dysfunction remains incomplete.

Episodic memory refers to the ability to encode, store, and retrieve information tied to personal experiences in time and space, and is a fundamental aspect of human cognition (Tulving & Markowitsch, 1998). This system relies on the coordinated operation of three interconnected processes: encoding, consolidation, and retrieval (Atkinson & Shiffrin, 1968). Encoding involves the transformation of perceptual information into neural representations. This process relies on the interplay between prefrontal executive systems guiding attention and strategic organization, and medial temporal lobe structures such as the hippocampus, entorhinal cortex, and parahippocampal gyrus, that bind sensory features into coherent episodes (Doss et al., 2023, Takehara-Nishiuchi, 2014). Consolidation refers to the stabilization and long-term storage of newly encoded memories. This process critically depends on hippocampal mechanisms and their interaction with neocortical regions (Mekbib and McDonough, 2025, Takehara-Nishiuchi, 2021). Retrieval involves accessing and reconstructing stored information, engaging both hippocampal processes responsible for reassembling fragmented sensory details, and prefrontal mechanisms guiding strategic search and source monitoring (Hainmueller and Bartos, 2020, Risius et al., 2018, Sander et al., 2021)

Although structural compromise of the hippocampus and medial temporal lobes has been consistently documented in HD (Johnson et al., 2021, Martinez-Horta et al., 2020, Rosas et al., 2008, Wilkes et al., 2023), episodic memory impairments in this disease have traditionally been attributed to executive dysfunction in terms of deficits in retrieval strategies and attentional encoding, rather than to primary hippocampal-based encoding or consolidation failures (El Haj et al., 2013, Martinez-Horta et al., 2020, Montoya et al., 2006). Consequently, the canonical model of memory dysfunction in HD posits a fronto-executive disorder with secondary mnemonic consequences. However, emerging evidence indicates that HD patients may also experience memory deficits more characteristic of medial temporal lobe pathology, involving disruption of encoding and consolidation processes (Carmichael et al., 2019, Glikmann-Johnston et al., 2019, Harris et al., 2019). Specifically, these studies report hippocampus-related impairments in spatial and episodic memory, where performance correlates with medial temporal volume loss, supporting the notion that consolidation and retrieval deficits in HD reflect additional medial temporal involvement. Despite these findings, systematic investigations into the nature and variability of episodic memory impairments in HD remain scarce. Moreover, the relationship between distinct memory profiles, neuroanatomical alterations, and biological markers of disease progression has not been fully elucidated

In the preset study, we hypothesized that episodic memory dysfunction in HD may be heterogeneous, reflecting the involvement of distinct neural systems and pathological mechanisms. Specifically, we posited that while many patients would exhibit a retrieval-dominant profile consistent with frontal-striatal disruption, a subgroup might display a memory impairment pattern indicative of medial temporal lobe degeneration.

To test this hypothesis, we conducted an in-depth analysis of episodic memory performance in HD using the Free and Cued Selective Reminding Test (FCSRT), a paradigm sensitive to disentangling encoding, consolidation, and retrieval processes. Additionally, we applied the Item-Specific Deficit Approach (ISDA) to quantify deficits across these stages of memory processing. We further examined the neural correlates of memory impairments through voxel-based morphometry (VBM) analysis of structural MRI data, and explored associations with plasma neurofilament light chain (NfL) levels, a biomarker of neuroaxonal damage.

## 2 Methods

### 2.1 Participants and assessments

We recruited a total sample of 44 symptomatic gene-mutation carriers (CAG > 39) from the outpatient clinic of the Movement Disorders Unit at Hospital de la Santa Creu i Sant Pau in Barcelona and 21 healthy controls. Demographic and clinical information, including age, gender, and education level, was documented. Motor symptom severity was evaluated using the Unified Huntington’s Disease Rating Scale − Total Motor Score (UHDRS-TMS) (Group, 1996). Functional abilities were measured through the Total Functional Capacity (TFC) (Group, 1996, Shoulson and Fahn, 1979). Patients were categorized into stages 2 or 3 based on the biological classification of the Huntington’s Disease Integrated Staging System (HD-ISS) (Tabrizi et al., 2022). In this study, patients classified as HD-ISS = 3 were required to meet the criteria for mild functional impairment according to the TFC scale. The CAG-age product (CAP score) was calculated as a measure of the cumulative impact of the CAG repeat expansion in the HTT gene over time. This measure was derived using age and CAG repeat length with the formula: age × (CAG − 33.66) (Warner et al., 2022).

Neither patients nor controls were allowed to have a history of any other neurological or psychiatric disorder, a prior traumatic brain injury with loss of consciousness, or an uncontrolled systemic disease. Patients with active substance use, psychotic and/or delusional symptoms were also excluded. Finally, individuals deemed by the medical team to be unable to understand or perform the tasks required for this study were excluded.

Global cognitive function was evaluated using the Parkinson’s Disease Cognitive Rating Scale (PD-CRS), which overcomes the sensitivity limitations of tools like the MMSE or MoCA when used in HD population (Horta-Barba et al., 2023, Martinez-Horta et al., 2020, Pagonabarraga et al., 2008). Despite the extensive knowledge regarding the utility of other cognitive measures focused on specific processes (e.g., SDMT) in HD, these measures fail to provide a comprehensive view of the cognitive status. Therefore, recognizing the need to globally assess patients' cognitive function, and based on its proved reliability, we selected the PD-CRS (Martinez-Horta et al., 2020). This test assesses overall cognition through nine subtests and prior research in HD has shown that the PD-CRS exhibits strong psychometric properties for classifying HD patients according to global cognitive status and to monitoring longitudinal changes (Horta-Barba et al., 2023, Martinez-Horta et al., 2020).

The protocol for the present study was reviewed and approved by the ethics committee of our institution. Participants provided written informed consent prior to their participation in the study. All procedures were conducted in accordance with the principles outlined in the 1964 Declaration of Helsinki and its subsequent amendments. The data that support the findings of this study are available from the corresponding author upon reasonable request.

#### 2.1.1 Assessment of verbal episodic memory

In the present study, the version A of the Spanish Free and Cued Selective Reminding Test (FCSRT) was used to evaluate verbal episodic memory performance (Buschke, 1984, Pena-Casanova et al., 2009). The administration of the FCSRT begins with a presentation phase where participants were introduced to a list of 16 words, each paired with a specific semantic category (e.g., “furniture − desk”). Participants were asked to repeat each word after its presentation to ensure proper encoding, and any word not recalled was repeated until the participant was able to accurately produce it. This was followed by three learning trials consisting of an initial free recall phase, during which participants attempted to recall as many words as possible without cues, followed by a cued recall phase for words not retrieved during free recall, using their respective semantic categories as prompts. To enhance encoding, a non-semantic interference task, such as a brief distraction activity, were introduced after each cued recall phase. After a 30-minute delay, a delayed recall phase was conducted, consisting of a free recall trial and a subsequent cued recall trial for words not retrieved in the free recall. This comprehensive procedure ensured both robust encoding and assessment of retrieval processes under free and cued conditions.

The selection of the FCSRT was based on the evidence regarding the strong psychometric properties of this test, including reliability and validity, for detecting memory impairments in conditions such as Alzheimer’s disease, mild cognitive impairment (MCI), and other disorders involving medial temporal lobe dysfunction, and because the FCSRT it is sensitive to the early stages of hippocampal atrophy (Grande et al., 2018, Grober et al., 2010, Teichmann et al., 2017). Moreover, the inclusion of semantic cues minimizes the impact of executive function deficits, language disorders, or attentional problems on memory performance, allowing a more targeted assessment of episodic memory and medial temporal lobe function (Buschke, 1984). The FCSRT has been successfully applied in a variety of neurological and psychiatric conditions (Girtler et al., 2022, Horta-Barba et al., 2020, Siquier and Andres, 2021).

#### 2.1.2 Item-Specific Deficit Approach

To further analyze the processes involved in performance on the FCSRT, in addition to the original scoring methodology, we also applied the Item-Specific Deficit Approach (ISDA) (Oltra-Cucarella et al., 2018, Wright et al., 2009). Instead of analyzing aggregated scores or overall performance, the ISDA evaluates the deficits at level of encoding, retention, or retrieval of specific items. This allows for the identification of patterns such as selective forgetting or impaired access to certain types of information. Moreover, the ISDA distinguishes between problems arising during the encoding phase (e.g., failure to adequately encode the item into memory) and those occurring during retrieval (e.g., inability to access the encoded information despite its presence in memory). This distinction is particularly relevant for understanding hippocampal and medial temporal lobe function.

The ISDA indices scores reflect worsening performance or greater severity as the index value increases. The ISDA indices were calculated follows (Oltra-Cucarella et al., 2018):

1. *Encoding Deficit Index (EncDI):* This index reflects the amount of information poorly encoded during the learning process. It is calculated as the sum of items in a list-learning test that were not recalled in more than half of the learning trials, divided by the total number of items. In the present study, this was obtained by summing the number of words recalled fewer than three times across the three learning trials and dividing by 16.
2. *Consolidation Deficit Index (ConsDI):* This index represents the amount of information recalled at least once during the learning trials, either through free or cued recall, that was not recalled during delayed recall, neither freely nor with cues. It is calculated as the sum of items recalled during the learning phase but not recalled in subsequent delayed recall trials, divided by the total number of items recalled at least once during the learning phase (via free or cued recall).
3. *Retrieval Deficit Index (RetDI):* This index measures the information encoded at least once during learning, either through free or cued recall, that is retrieved with cues during delayed free recall. It is calculated as the sum of items recalled during the learning phase but inconsistently recalled in the delayed recall task, divided by the total number of items recalled at least once during the learning phase.

For the Consolidation and Retrieval indices, normalization by the number of items recalled at least once during learning ensures that these indices reflect the proportional efficiency of each process, rather than absolute recall capacity. This approach, established in the original ISDA formulation, minimizes confounding by interindividual differences in overall learning performance.

### 2.2 Neuroimaging acquisition and preprocessing

High-resolution T1-weighted MRI images (voxel size ≈ 0.9 × 0.9 × 1 mm) were obtained on a 3 T Philips Achieva scanner using an MPRAGE sequence (TR/TE = 12.65/7.08 ms, flip angle = 8°, field of view = 23 cm, matrix = 256 × 256, slice thickness = 1 mm). During preprocessing, all images were normalized and resampled to an isotropic voxel resolution of 1 mm^3^ as part of the standard VBM pipeline implemented in SPM12. Standard voxel-based morphometry (VBM) procedures were performed using the SPM12 software package (https://www.fil.ion.ucl.ac.uk/spm). Gray matter volume (GMV) tissue probability maps were generated from the T1-weighted scans and subsequently normalized to the Montreal Neurological Institute (MNI) space using the DARTEL algorithm. To account for inter-individual variability, the normalized GMV maps were smoothed with an isotropic Gaussian filter with a full-width at half-maximum (FWHM) of 8 mm in all dimensions.

### 2.3 Biosamples collection and processing

Blood draw was performed on-site, collected in EDTA tubes and centrifuged at 2000*g* for 10 min, and the plasma aliquoted in polypropylene tubes and frozen at 80 °C until analysis according to international consensus recommendations. Neurofilament light (NfL) were measured with the Simoa Human NF-light Advantage kit (Catalogue # 104073) using the Single Molecule Array (Simoa) technology (Simoa; Quanterix, Lexington, MA, United States) in the SR-X Biomarker detection system by following the manufacturer’s instructions. Briefly, the calibration curve, control samples, inter-assay control (pooled plasma with known concentration) and unknown samples in duplicate were loaded onto a 96-well plate. Samples were incubated with antibody coated paramagnetic beads and biotinylated antibody detector simultaneously. After a wash, streptavidin-conjugated β-galactosidase (SBG) reagent was added to bind the biotinylated antibodies, leading to the SBG enzyme labeling of the capture of NfL. Once in the SR-X platform, the beads were resuspended in resorufin β-D-galactopyranoside (RGP) reagent, transferred to the Simoa disk and sealed. The NfL proteins captured by the antibody coated paramagnetic beads and labeled with the SBG reagent hydrolyze the RGP substrate to produce a fluorescent signal. The fluorescent signal values generated from the calibration curve of known concentrations were fit using a 4-parameter logistic curve that was used to calculate the unknown and control samples concentrations. All the samples were measured in duplicate and had an intra-assay coefficient of variation (CV) < 20 %.

### 2.4 Statistical analysis

Non-imaging analyses were performed using SPSS v.23 (IBM, Armonk, NY). Group differences in demographic, clinical, and cognitive variables between HD patients and healthy controls (HC) were assessed using independent-samples t-tests for continuous variables and χ^2^ tests for categorical variables.

For the FCSRT, raw and scaled scores were analyzed alongside the ISDA indices (Encoding, Consolidation, and Retrieval Deficit Indices). Scaled scores were derived from Spanish normative data (Pena-Casanova et al., 2009), and the equivalent z-values were computed following standard neuropsychological conventions.

Between-group effects (HD vs. HC) on memory performance were further explored using mixed-model ANOVAs with “recall type” (free vs. cued) and “time” (immediate vs. delayed) as within-subject factors and “group” as the between-subject factor.

To determine which memory variables best discriminated between HD and controls, binary logistic regressions were conducted with FCSRT and ISDA measures as predictors, followed by ROC curve analyses to evaluate diagnostic accuracy.

Beyond these primary comparisons, we examined intra-cohort heterogeneity by dividing HD participants into two subgroups (HD-1 and HD-2) according to their delayed total recall scaled score on the FCSRT. This classification followed the NEURONORMA normative criterion, where a scaled score ≤ 6 (≈ z ≤ –1.28) represents performance below the 9th percentile and is considered impaired (Pena-Casanova et al., 2009). Analyses comparing HD-1 and HD-2 followed the same statistical procedures as those used for the HD vs. HC contrasts.

For voxel-based morphometry (VBM) analyses, voxel-wise gray matter volume (GMV) comparisons were conducted using SPM12 within a general linear model, controlling for age, sex, and education. Additional GLMs explored voxel-wise correlations between GMV and memory measures (FCSRT and ISDA indices) within the HD group, including CAP score, age, and education as covariates. Statistical significance was set at p < 0.05, FWE-corrected at the cluster level.

Analyses were structured across three independent domains—neuropsychological, neuroimaging, and plasma biomarker—each addressing a specific hypothesis. Given their conceptual independence, no global multiple-comparison correction was applied across domains. Behavioral and plasma analyses were considered exploratory and hypothesis-driven.

## 3 RESULTS

The sample of patients with HD consisted of forty-four participants divided into 54.5 % women and 45.5 % men, with a mean age of 53.27 ± 10.9 years, an average education level of 13.57 ± 3.6 years, a mean CAG repeat length of 43.93 ± 3.48, a mean UHDRS-TMS score of 38.09 ± 23, a mean Total Functional Capacity (TFC) score of 9.86 ± 2.8, and an average PD-CRS score of 76.49 ± 19. The PD-CRS and TFC scores indicate that, on average, participants had a global cognitive score within the range of mild cognitive impairment and were in the early to intermediate stages of the disease.

The control group (HC) consisted of twenty-one participants divided into 52.4 % women and 47.6 % men, with a mean age of 48.86 ± 12.6 years, an average education level of 14.43 ± 3.62 years, full functional capacity (13 points in all cases), and a mean PD-CRS score of 106.62 ± 13.2. The cognitive and functional scores of the control group indicated that participants had a globally normal cognitive state and full functional capacity.

Independent samples t-tests showed, as illustrated in Table 1, that there were no significant differences between HC and HD patients in terms of age and years of education. Pearson’s Chi-square test demonstrated no significant differences between groups in gender distribution [χ^2^ = 0.027; p = 0.540]. As expected, highly significant differences were observed between patients and controls in global cognitive status based on the PD-CRS [t(65) = 6.5; p < 0.0001], motor symptom severity based on the UHDRS-TMS [t(65) = 10.9; p < 0.0001], functional status based on the TFC [t(65) = 7.2; p < 0.0001], as well as on NfL plasma levels [t(65) = 10.73; p < 0.0001], all with large effect sizes (d > 0.8).

**Table 1:** Clinical and sociodemographic characteristics of the sample.

	HC (n = 21)	HD (n = 44)	*p*	*d*
Age	48.86 ± 12.6	53.27 ± 10.9	0.152	0.374
Education (years)	14.43 ± 3.6	13.57 ± 3.6	0.378	0.238
Sex (% woman)	52.4 %	54.5 %	0.540	−
CAG	−	43.93 ± 3.4	−	−
CAP score	−	516.47 ± 96.7	−	−
UHDRS-TMS	1 ± 0.4	38.09 ± 23	<0.0001	2.280
TFC	13	9.86 ± 2.8	<0.0001	1.584
PD-CRS	106.62 ± 13.2	76.49 ± 19	<0.0001	1.841
NfL	5.6 ± 3.4	26.1 ± 11.7	<0.0001	2.379

UHDRS-TMS: Unified Huntington’s Disease Rating Scale – Total Motor Score, TFC: Total Functional Capacity, PD-CRS: Parkinson’s Disease – Cognitive Rating Scale, NfL: Neurofilament light chain plasma levels.

### 3.1 Verbal episodic memory performance

**Fig. 1:**
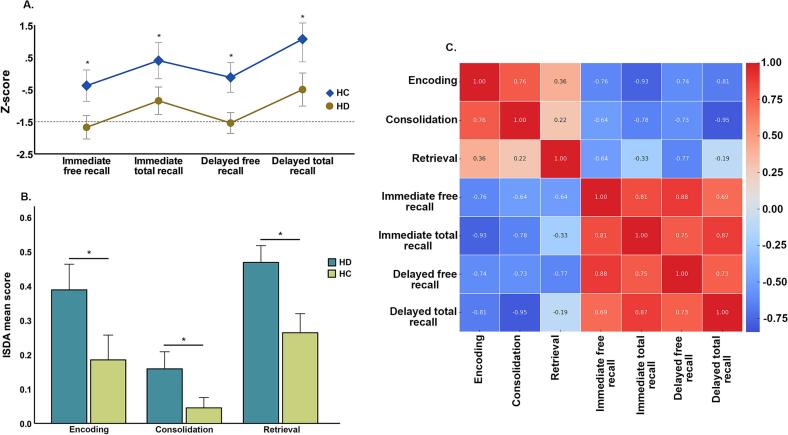
FCSRT and ISDA performance between HC and HD participants. In section "A," the graphical representation of the mean (SEM) distribution of standardized FCSRT scores in both groups is displayed. The dashed gray line is positioned at z = 1.5 (cutoff z ≤ 1.28). It can be observed that in the controls (blue line), performance is above the cutoff in all cases, whereas in the patients, performance in immediate and delayed free recall falls below the cutoff. In section "B," a bar chart illustrates the mean (SEM) scores obtained for the various indices across groups. Section “C” depicts the correlation heat-map between the different memory-related metrics. Significant differences (p < 0.05) are marked with "*".

The three-way mixed-effects ANOVA with recall type (free vs. cued) and time (immediate vs. delayed) as within-subjects factors, and group (HD vs. HC) as a between-subjects factor revealed a significant main effect of group [F(1, 244) = 64.32, p < 0.001], with patients performing significantly worse than controls across all conditions, and a significant main effect of time [F(1, 244) = 801.08, p < 0.001] showing better performance in delayed recall compared to immediate recall. A significant main effect of recall type [F(1, 244) = 307.52, p < 0.001] was also observed, showing that total recall scores were higher than free recall scores across groups (Fig. 1).

Significant two-way interactions were found between group and time [F(1, 244) = 9.70, p = 0.002], and between group and recall type [F(1, 244) = 4.73, p = 0.031], suggesting that group differences varied according to the retrieval phase and that the magnitude of facilitation (cued vs. free) differed between groups. The time × recall type interaction was significant [F(1, 244) = 83.02, p < 0.001], while the three-way interaction was not [F(1, 244) = 0.18, p = 0.673]. As seen in Table 2, post-hoc pairwise comparisons confirmed that patients performed significantly worse than controls across all conditions and that differences remained statistically significant after Bonferroni correction. A parallel three-way ANOVA was conducted on standardized z-scores, yielding similar results. A significant main effect of group [F(1, 244) = 56.77, p < 0.001] and of recall type [F(1, 244) = 32.69, p < 0.001] was observed, while time and all interactions were non-significant. Post-hoc comparisons using z-scores confirmed significant group differences across conditions. Importantly, based on standardized z-scores, performance in the HD group was clinically impaired (z ≤ -1.28) in immediate free recall (mean = −1.72) and delayed free recall (mean = −1.58), while scores for immediate and delayed total recall remained within the low-normal range (mean = −0.87 and mean = −0.52, respectively)

**Table 2:** FCSRT and ISDA Indices performance.

		HC (n = 21)	HD (n = 44)	*p*	*d*
Immediate free recall		28.05 ± 7.2	17.79 ± 8.4	<0.0001	1.311
	*Z-score*	−0.44 ± 1.1	−1.67 ± 1.2	<0.0001	1.068
Immediate total recall		44.24 ± 4	37.86 ± 7.4	<0.0001	1.072
	*Z-score*	0.33 ± 1.3	−0.85 ± 1.4	0.002	0.873
Delayed free recall		11 ± 2.7	6.02 ± 3.5	<0.0001	1.593
	*Z-score*	−0.18 ± 1	−1.54 ± 1.1	<0.0001	1.293
Delayed total recall		15.19 ± 1.1	12.83 ± 3.1	<0.0001	1.014
	*Z-score*	1 ± 1.6	−0.5 ± 1.6	0.001	0.937
Forgetting index		0.98 ± 0.03	0.90 ± 0.14	0.001	0.790

ISDA					
	Encoding	0.18 ± 0.16	0.38 ± 0.24	<0.0001	0.980
	Consolidation	0.04 ± 0.06	0.15 ± 0.16	<0.0001	0.910
	Retrieval	0.26 ± 0.12	0.46 ± 0.16	<0.0001	1.414

Values represent mean ± standard deviation (SD) for raw and Z-scores.

To further characterize the specific memory processes underlying the FCSRT performance, we analyzed group differences across the ISDA indices. Independent-samples t-tests revealed that HD exhibited significantly greater encoding [t(62) = 3.92, p = 0.0002], consolidation [t(62) = 3.89, p = 0.0002] and retrieval deficits [t(62) = 5.50, p < 0.001].

We explored the relationships between ISDA indices and raw FCSRT scores. Significant positive correlations were observed between encoding and consolidation deficits (r = 0.65, p < 0.001), and between encoding and retrieval deficits (r = 0.32, p = 0.004). No significant correlation was found between consolidation and retrieval deficits (r = 0.21, p = 0.081). Regarding the relationship between ISDA indices and FCSRT performance, higher deficits in encoding and consolidation were strongly associated with lower scores across all FCSRT measures (all p < 0.001). Retrieval deficits were also significantly associated with worse FCSRT performance, but the association with delayed total recall was not statistically significant.

Multiple linear regression analyses were conducted to examine the contribution of clinical variables to memory performance. Predictors included age, CAG repeat length, UHDRS-TMS, PD-CRS total score, and plasma NfL. All regression models for FCSRT outcomes were statistically significant: immediate free recall [R^2^ = 0.63, F(5, 34) = 11.59, p < 0.001], immediate total recall [R^2^ = 0.54, F(5, 34) = 8.00, p < 0.001], delayed free recall [R^2^ = 0.61, F(5, 34) = 10.59, p < 0.001], and delayed total recall [R^2^ = 0.52, F(5, 34) = 7.45, p < 0.001]. In each of these models, the PD-CRS total score was the only significant individual predictor, with higher cognitive scores associated with better memory performance (all β ≈ 0.09 to 0.12, all p < 0.005). Neither age, CAG repeat length, UHDRS-TMS, nor NfL levels significantly predicted FCSRT performance. Separate linear regression models were estimated to explore the contribution of clinical variables to each of the ISDA indices. The model predicting encoding deficits was statistically significant [R^2^ = 0.50, F(5, 34) = 6.82, p < 0.001] with greater deficits significantly associated with lower PD-CRS scores (B = −0.0074, p = 0.006, 95 % CI [−0.012, −0.002]). The model predicting consolidation deficits was also significant [R^2^ = 0.44, F(5, 34) = 5.30, p = 0.001], with PD-CRS again emerging as the only significant predictor (β = −0.0053, p = 0.005, 95 % CI [−0.009, −0.0017]).

To identify which memory processes best discriminated between HD and HC, we conducted a binary logistic regression using standardized FCSRT raw scores (immediate and delayed free and total recall). The model was statistically significant [χ^2^(4) = 26.07, p < 0.0001], with a pseudo-R^2^ of 0.33. Among the predictors, only delayed free recall significantly predicted group membership (B = −1.96, OR = 0.14, 95 % CI [0.03, 0.74], p < 0.05), indicating that lower performance in delayed free recall was strongly associated with a higher probability of being classified as HD. The receiver operating characteristic (ROC) curve analysis for this model showed an area under the curve (AUC) of 0.84, with a 95 % confidence interval (CI) of [0.76, 0.96], indicating good discriminative ability between HD and HC.

The logistic regression model using ISDA indices was also statistically significant [χ^2^(3) = 13.49, p < 0.00001], with a pseudo-R^2^ of 0.337. Among the predictors, only the retrieval deficit index significantly predicted group membership (B = 1.44, OR = 4.21, 95 % CI [1.68, 10.59], p < 0.01), indicating that greater retrieval deficits were associated with higher odds of belonging to the HD group. The ROC curve analysis for this model yielded an AUC of 0.87, with a 95 % CI of [0.77, 0.95], reflecting a very good level of discriminative capacity.

### 3.2 Divergent patterns of memory performance within the HD sample

Considering the objectives and hypotheses of the study, we identified cases scoring below z ≤ 1.28 in the FCSRT delayed total recall in both the patient and controls. In the HC group, no one case scored ≤ 1.28. In contrast, in the HD group, 14 cases (31.8 % of the sample), obtained a score below the standardized normality in the FCSRT delayed total recall. Accordingly, this approach revealed that, within the HD group, there was a subgroup of patients with significantly impaired performance on the FCSRT delayed recall due to a lack of significant benefit from the facilitation using semantic cues as reflected by the impaired performance on de delayed total recall. Subsequently, the group of patients exhibiting this pattern of performance of z ≤ 1.28 on the FCSRT delayed total recall was labeled as HD-1, otherwise as HD-2. Then, we conducted a comparative analysis between them, which demonstrated that there were no differences in age, years of education, sex, CAG repeat length, CAP score or NfL plasma levels. In contrast, significant differences between groups were found for UHDRS-TMS [t(44) = 2.71; p = 0.010], TFC [t(44) = 2.9; p = 0.009], and global cognitive status as assessed with the PD-CRS [t(44) = 3.52; p = 0.001]. (See Table 3)

**Table 3:** FCSRT and ISDA Indices performance between HD-1 and HD-2.

		HD-1 (n) = 14)	HD-2 (n = 30)	*p*	*d*
Age		52.94 ± 11.3	53.46 ± 10.8	0.513	0.047
Education (years)		13 ± 4.2	13.89 ± 3.3	0.443	0.235
Sex (% woman)		55	45	0.277	
CAG		44.31 ± 2.2	43.22 ± 3.3	0.298	0.388
CAP score		509.06 ± 84.6	479.37 ± 70.5	0.229	0.381
UHDRS-TMS		54.38 ± 20.7	28.79 ± 18.9	<0.0001	1.291
TFC		8.13 ± 3.4	10.8 ± 1.9	0.009	0.969
PD-CRS		61.53 ± 11.4	84.50 ± 17.5	<0.0001	1.555
NfL		24.87 ± 7.9	23.68 ± 11.3	0.723	0.122
Immediate free recall		11.71 ± 3.7	20.82 ± 8.5	<0.0001	1.389
	*Z-score*	−2.62 ± 0.3	−1.21 ± 1.2	<0.0001	1.612
Immediate total recall		30.43 ± 5.3	41.57 ± 5.2	<0.0001	2.121
	*Z-score*	−2.02 ± 0.6	−0.28 ± 1.3	<0.0001	1.718
Delayed free recall		3.29 ± 2.3	7.39 ± 3.2	<0.0001	1.471
	*Z-score*	−2.54 ± 0.4	−1.05 ± 0.9	<0.0001	2.139
Delayed total recall		9.43 ± 2.9	14.54 ± 1.3	<0.0001	2.273
	*Z-score*	−2.21 ± 0.6	0.32 ± 1.4	<0.0001	2.349
Forgetting index		0.77 ± 0.1	0.96 ± 0.7	<0.0001	0.38
ISDA					
	Encoding	0.60 ± 0.2	0.27 ± 0.2	<0.0001	1.65
	Consolidation	0.32 ± 0.1	0.07 ± 0.08	<0.0001	2.760
	Retrieval	0.44 ± 0.14	0.47 ± 0.16	0.562	0.199

**Fig. 2:**
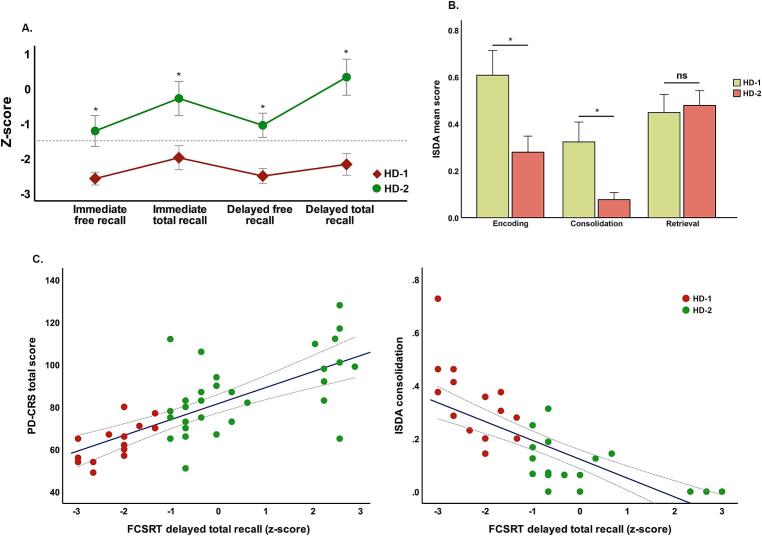
FCSRT and ISDA performance between HD-1 and HD-2 participants. In section "A," the graphical representation of the mean (SEM) distribution of standardized FCSRT scores in both groups is displayed. In the HD-2 group (green line), performance is above the cutoff in almost all cases, with the lowest performance observed in the free recall sections. In the HD-1 group, performance is below the cutoff at all points, with a notable lack of improvement in long-term cued recall. In section "B," a bar chart illustrates the mean (SEM) scores obtained for the various indices across groups. The evident differences in encoding and consolidation deficits in HD-1 can be observed, while no differences are present in the retrieval index. In section “C” the scatterplots on the left margin illustrate the association between total free recall performance and total PD-CRS scores. It can be observed that lower PD-CRS scores are associated with poorer memory performance, with most cases classified as HD-1 (red circles) occupying the extreme of poorest performance. On the right margin, the association between the deficient consolidation index and total free recall is displayed. Again, most cases classified as HD-1 are positioned at the extreme end of the graph. Significant differences (p < 0.05) are marked with "*".

Independent-samples t-tests comparing raw FCSRT scores revealed robust and statistically significant differences across all measures. Thus, HD-1 exhibited significantly lower immediate free recall scores [t(32.6) = 4.80, p < 0.001], immediate total recall scores [t(30.8) = 6.39, p < 0.001], delayed free recall scores [t(28.3) = 4.70, p < 0.001], and delayed total recall scores [t(33.4) = 6.11, p < 0.001], compared to HD-2. Analyses of standardized z-scores yielded a similar pattern. HD-1 showed significantly lower immediate free recall z-scores [t(27.4) = 5.58, p < 0.001], immediate total recall z-scores [t(27.8) = 5.64, p < 0.001], delayed free recall z-scores [t(26.9) = 7.06, p < 0.001], and delayed total recall z-scores [t(25.3) = 8.24, p < 0.001] compared to HD-2. Importantly, z-scores in HD-1 fell below the clinical threshold for significant impairment (z ≤ -1.28), indicating widespread and clinically meaningful episodic memory deficits across both free and cued conditions, as well as across immediate and delayed retrieval phases(see Fig. 2).

A binary logistic regression analysis was conducted to examine which memory processes, indexed by ISDA deficits, was better associated with subgroup classification. The model was statistically significant [χ2(3) = 33.33, p < 0.001], with a pseudo-R2 of 0.623, indicating that approximately 62 % of the variance in group classification was explained by the ISDA indices. Among the predictors, the standardized consolidation deficit index significantly predicted group membership, with greater consolidation deficits associated with higher odds of belonging to the HD-1 group (B = 3.25, OR = 25.85, 95 % CI [3.40, 196.63], p = 0.019). The model’s discriminative ability was excellent, as reflected by an area under the receiver operating characteristic (ROC) curve (AUC) of 0.96, with a 95 % confidence interval (CI) ranging from 0.89 to 1.00.

### 3.3 Analysis of plasma neurofilament light levels and memory performance profiles

To examine whether neurodegeneration markers were associated with memory performance profiles, plasma NfL levels were compared between patients classified as HD-1 and HD-2. An independent-samples *t*-test revealed no significant difference in NfL levels between groups [t(26.9)  = −0.26, p = 0.794]. Within-group Pearson correlation analyses revealed no significant associations between NfL levels and FCSR delayed total recall or ISDA consolidation deficits.

### 3.4 Neuroimaging analysis

The voxelwise group comparison performed between HD and HC, revealed a significant pattern of GMV decrease in HD, with extremely pronounced effects in the basal ganglia [t = 15.3; ^k^E = 20104; FWE p < 0.05], and in multiple cortical and subcortical regions, including the parietal [t = 7.72; ^k^E = 615; FWE p < 0.05], the mid [t = 7.07; ^k^E = 2228; FWE p < 0.05] and superior occipital [t = 6.70; ^k^E = 1426; FWE p < 0.05], the frontal [t = 6.95; ^k^E = 281; FWE p < 0.05], and temporal [t = 6.51; ^k^E = 149; FWE p < 0.05] areas, among several others (figure in supplementary data 1).

**Fig. 3:**
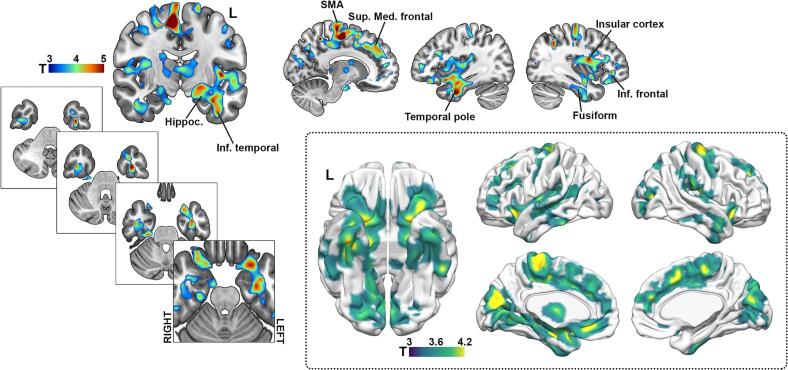
GMV correlates of memory performance in HD patients. The figure depicts de GMV correlates of FCSRT delayed total recall. For depicting purposes p-value was set at p<0.001; k=20.

Since we aimed to avoid effects mediated by collinearity between metrics closely related to disease progression and memory performance, and because we also sought to exclude anatomical differences associated with normal variability in GMV that could influence memory performance, we focused the analysis of imaging correlates on the HD group. The voxelwise regression analysis (Fig. 3) showed several associations between GMV and memory performance as measures using the FCSRT delayed total recall. The more prominent associations surviving FWE correction at a cluster level involved the SMA [t = 7.60; ^k^E = 21156; FWE p < 0.0001], a left-lateralized big cluster extending from the left medial orbital, to the left hippocampus, amygdala, fusiform gyrus and inferior temporal, entorhinal cortex and temporal pole [t = 5.48; ^k^E = 20657; FWE p < 0.0001], and to lesser extent, few right-lateralized clusters in the right inferior frontal gyrus extending to the right insular cortex [t = 5.23; ^k^E = 13652; FWE p < 0.0001].

#### 3.4.1 Imaging differences between HD groups

**Fig. 4:**
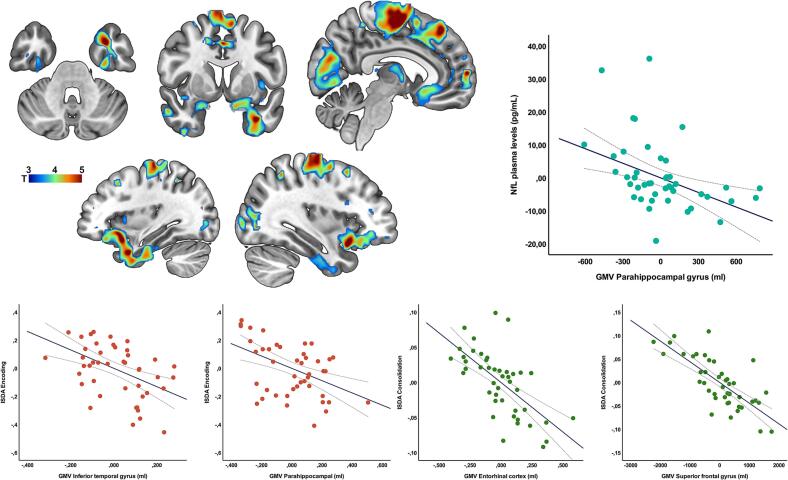
GMV differences between HD-1 and HD-2 groups. The figure depicts de GMV differences between HD-1 and HD-2 groups. For depicting purposes p-value was set at p<0.001; k=20. Scatter-plots shows results from linear regression analysis between specific GMV clusters, NfL and ISDA indices.

In a first approach we conducted a voxelwise group comparison between HC > HD-1 and between HC > HD-2. Subsequently, a voxelwise group comparison was performed between HD-1 and HD-2. The first analysis showed decreased GMV in the HD-2 group compared to HC bilaterally in all the regions comprising the basal ganglia [t = 11.63; ^k^E = 18268; FWE p < 0.05], the inferior [t = 5.96; ^k^E = 1700; FWE p < 0.05] and superior [t = 5.52; ^k^E = 2686; FWE p < 0.05] parietal, the superior occipital [t = 5.78; ^k^E = 3398; FWE p < 0.05], and the supramarginal gyrus [t = 4.76; ^k^E = 1001; FWE p < 0.05]. The comparison between HC and HD-1 showed much more dramatic GMV differences also expressed bilaterally at the level of the basal ganglia and extending to the whole brain [t = 16.18; ^k^E = 255319; FWE p < 0.05] (Figure in supplementary data 2). The voxelwise group comparison between HD-1 and HD-2 revealed a significant reduction of GMV in HD-1 compared to the HD-2 (FWE corrected at cluster level) in the right and specially left mid temporal pole [t = 5.37; ^k^E = 7008; FWE p < 0.0001], and medial temporal pole extending to the left fusiform gyrus and parahippocampal gyrus [t = 4.85; ^k^E = 4472; FWE p = 0.006], and entorhinal cortex [t = 3.97; ^k^E = 201; FWE p = 0.008], together with an evident pattern of more widespread whole-brain atrophy also involving, among others, the thalamus and occipital cortex (See Fig. 4).

A linear regression analysis was used to explore the contribution of structural integrity on each of these regions to the specific ISDA indices. Accordingly, we conducted a multivariate regression analysis for each ISDA index as dependent variables, and all the previously identified GMV clusters and nuisance variables as independent variables. This analysis showed a negative association between GMV integrity in the cluster comprising the left inferior temporal pole, parahippocampal and inferior temporal gyrus and the encoding index (β = −0.349; t = 2.63; p = 0.011). Performance on the consolidation index showed several significant negative associations with structural integrity at the level of the left entorhinal cortex (β = −0.501; t = 3.01; p = 0.005), the left middle temporal (β = −0.553; t = 2.8; p = 0.008), and superior temporal gyrus (β = −0.434; t = 3.1; p = 0.004), and the left superior frontal (β = −0.540; t = 3.3; p = 0.002). None of the identified clusters showed a significant association with the retrieval index.

Although no significant differences in plasma NfL levels were found between memory performance subgroups, we further explored whether NfL levels were associated with brain structural integrity across the cohort of patients. To this end, multiple linear regression analyses were conducted. The results showed that higher NfL levels were significantly associated with lower volumes in several cortical and subcortical regions. In particular, a significant negative association was found with the left middle temporal gyrus volume (β = −0.96, p = 0.034, R^2^ = 0.15) and left hippocampal volume (β = −0.12, p = 0.046, R^2^ = 0.11).

## 4 DISCUSSION

The present study sought to comprehensively characterize episodic memory in HD using the Free and Cued Selective Reminding Test (FCSRT) combined with process-specific analyses via the Item-Specific Deficit Approach (ISDA). Additionally, we aimed to explore the structural and biomarker correlates of memory impairment to better understand the neurobiological substrates underlying these deficits in HD.

Our findings revealed robust episodic memory impairments in HD patients compared to healthy controls, affecting both free and cued recall measures. The most striking differences were observed in free recall, with performances falling clearly into the clinical impairment range for both immediate and delayed conditions. Although deficits were also present in cued recall measures, mean performances for these variables remained, on average, within the range of normality, suggesting a partial preservation of learning processes when external retrieval support was provided. This pattern indicates that spontaneous retrieval mechanisms are significantly compromised in HD, but that memory traces can still be accessed when aided by semantic cueing(El Haj et al., 2013, Montoya et al., 2006).

The analysis of ISDA indices reinforced this interpretation. While patients showed mild impairments across encoding, consolidation, and retrieval processes, the retrieval index remained the most critical discriminator between HD and controls. These results are consistent with models proposing that memory deficits in HD are predominantly mediated by frontal-executive dysfunction, where strategic access to stored information is disproportionately affected compared to encoding or storage(Oltra-Cucarella et al., 2018). In this regard, we consider it important to emphasize the value of using approaches such as the ISDA, which allow a more fine-grained decomposition of the specific processes involved in episodic memory formation. This level of analysis helps to uncover subtle anomalies that may remain undetected in this population when relying on more traditional or global memory measures.

At the clinical level, poorer memory performance was significantly associated with lower global cognitive status as measured by the PD-CRS, whereas no significant relationships emerged with motor severity or plasma NfL levels. This pattern indicates that episodic memory dysfunction in HD is more closely related to overall cognitive integrity than to general neurodegeneration. It likely reflects selective disruption of strategic brain regions critical for episodic memory, particularly within medial temporal and associated cortical networks. The use of the PD-CRS, a validated and comprehensive measure of global cognition in HD, complemented the specific memory assessments and enabled the interpretation of performance profiles within an integrated cognitive framework.

Beyond the global comparison between HD patients and healthy controls, a key contribution of this study was the identification of two distinct cognitive subgroups within the HD cohort, based on memory performance profiles. Approximately one-third of patients (HD-1) exhibited a pervasive memory impairment pattern, characterized by deficits not only in free recall but also in cued recall measures. In these cases, performance across all FCSRT components fell below clinical thresholds, and critical analysis of the ISDA indices revealed that the predominant impairment in this subgroup was related to deficits in memory consolidation, rather than encoding or retrieval. Accordingly, contrasting to the broader HD group, where retrieval difficulties were the dominant process affected, patients in HD-1 exhibited a striking failure to consolidate new information over time, resulting in severe impairments even when semantic cues were provided. These findings suggest that in this subgroup, neurodegenerative processes impact medial temporal structures more profoundly, disrupting the stabilization of newly encoded information into durable memory traces(Dickerson and Eichenbaum, 2010, Epelbaum et al., 2018, Takehara-Nishiuchi, 2014).

Clinical characterization of the subgroups revealed that patients in the HD-1 group had significantly worse cognitive performance as measured by PD-CRS, as well as greater motor and functional impairments. However, demographic variables, CAG repeat length or CAP score did not differ between groups. Importantly, plasma NfL levels were also comparable between subgroups, suggesting that traditional global markers of neurodegeneration do not fully capture the distinct neurocognitive phenotypes emerging in HD. Instead, our findings highlight that the presence of severe memory consolidation deficits reflects a more aggressive cognitive trajectory, independent of expansion size or general neuronal injury markers.

Neuroimaging analyses provided convergent evidence for the neuroanatomical underpinnings of the identified memory phenotypes. Across the whole HD cohort, total free recall performance correlated with gray matter volume in regions traditionally associated with episodic memory, including the left temporal pole, medial and inferior temporal cortices, entorhinal cortex, insular cortex, and fusiform gyrus(Dickerson and Eichenbaum, 2010, Epelbaum et al., 2018). These findings are consistent with the literature implicating medial and lateral temporal structures, along with frontal regions, in supporting the encoding, consolidation, and retrieval of episodic memories(Epelbaum et al., 2018). Although no direct correlation emerged between memory scores and plasma NfL levels, we observed that NfL concentrations were significantly associated with the degree of gray matter atrophy in these memory-related clusters.

When comparing the two patient subgroups, HD-1 exhibited widespread cortical atrophy, with significant volume reductions particularly in the left temporal pole, medial temporal cortex, parahippocampal gyrus, fusiform gyrus, and entorhinal cortex. This pattern of structural degeneration mirrors the functional deficits observed at the neuropsychological level, supporting the conclusion that in this subset of HD patients, memory impairments reflect not only retrieval difficulties but also profound disruption of memory consolidation processes(Dickerson and Eichenbaum, 2010, Epelbaum et al., 2018). Importantly, this pattern closely resembles the structural and functional profiles observed in amnestic syndromes secondary to medial temporal lobe pathology, suggesting that a subset of HD patients may evolve toward a distinct neurodegenerative trajectory affecting medial temporal systems more prominently(Martinez-Horta et al., 2023, Martinez-Horta et al., 2024, Oltra-Cucarella et al., 2018).

The absence of significant differences in plasma NfL levels between patient subgroups, despite clear evidence of greater neurodegenerative burden on MRI, should be interpreted with caution given the modest sample size and the corresponding limitations in statistical power. Nevertheless, the pattern observed is consistent with current knowledge regarding the biological specificity of NfL. This protein primarily reflects axonal injury, particularly within large-caliber myelinated fibers, and may therefore be less sensitive to focal gray matter degeneration or synaptic dysfunction in medial temporal structures critical for episodic memory. Previous studies in HD and other neurodegenerative disorders have shown that plasma NfL correlates more robustly with global white matter loss than with regional cortical atrophy, supporting this interpretation(Backstrom et al., 2020, Barro et al., 2020, Thompson et al., 2003). It is also plausible that NfL levels plateau or stabilize during the symptomatic stages represented in our cohort, when neuronal loss has already occurred, reducing sensitivity to subtle regional differences. Thus, while these explanations remain hypothesis-based, they are grounded in established empirical evidence and align with current models of the temporal and anatomical dynamics of NfL release in HD. The identification of a subgroup presenting with amnestic-like memory deficits, alongside medial temporal atrophy, challenges the traditional conception of HD-related memory dysfunction as being purely frontostriatal in nature. Instead, our findings underscore the heterogeneity of cognitive impairment in HD, suggesting that different patterns of neurodegeneration, and possibly, different underlying biological mechanisms, may contribute to clinical variability. These findings reinforce the notion that episodic memory impairments in HD, while often reflecting the prototypical frontal or frontostriatal dysfunction, can also involve significant disruptions in encoding and consolidation processes due to medial temporal and hippocampal degeneration(Carmichael et al., 2019, El Haj et al., 2013, Glikmann-Johnston et al., 2019, Harris et al., 2019). Importantly, such memory alterations are not universal among mutation carriers, nor can they be consistently explained by greater CAG repeat expansions or CAP scores(Martinez-Horta et al., 2023, Martinez-Horta et al., 2024). Instead, our results support the idea that HD may present with distinct clinical profiles, shaped by factors beyond the primary genetic mutation, and that these profiles manifest in specific cognitive deficits, such as episodic memory dysfunction. In this context, considering recent advances in understanding the mechanisms that modulate neurodegeneration in HD, notably, somatic instability among others, it is plausible that these secondary processes contribute significantly to the clinical heterogeneity observed (Genetic Modifiers of Huntington's Disease Consortium, 2019, Monckton, 2021, Scahill et al., 2025). Future research will be crucial to elucidate how such mechanisms influence not only disease onset but also the development of divergent cognitive trajectories within HD.

Despite these robust findings, several limitations of the present study should be acknowledged. First, although our sample size was adequate for detecting group differences and characterizing memory profiles, larger multicentric cohorts would allow finer-grained stratification and enhance the generalizability of the findings. Second, although plasma NfL provided important information regarding overall neurodegeneration, it may not fully capture the region-specific pathological processes underlying cognitive decline. Moreover, plasma quantification may be less sensitive than cerebrospinal fluid (CSF) measurements for detecting subtle or localized neuronal damage, particularly within critical memory-related structures. Future studies should consider incorporating additional biomarkers such as tau, GFAP, or markers of synaptic dysfunction, and explore CSF-based analyses to better delineate the biological substrates of episodic memory impairment in HD. Third, longitudinal designs are necessary to determine whether the identified cognitive subgroups represent distinct disease trajectories or different stages within a shared continuum. Nevertheless, the study’s strengths, including the use of process-specific memory analyses, multimodal integration of cognitive, structural, and biomarker data, and the identification of clinically meaningful cognitive phenotypes, provide valuable insights into the heterogeneity of cognitive decline in HD.

In conclusion, our study expands evidence that episodic memory dysfunction in HD encompasses a spectrum ranging from isolated retrieval deficits to profound impairments in memory consolidation, associated with medial temporal atrophy. This heterogeneity underscores the need for more nuanced clinical assessments and interventions, tailored to the specific cognitive profiles of patients. Furthermore, recognizing these distinct memory profiles could enhance our understanding of disease mechanisms and inform the development of targeted therapeutic strategies. Ultimately, appreciating the complexity and variability of cognitive decline in HD will be critical for advancing personalized medicine approaches in this population.

## Funding source

The present study was funded from Fondo de Investigaciones Sanitarias (FIS) from the Instituto de Salud Carlos III (Grant number: PI21/01758) and Fondos FEDER.

## Declaration of generative AI and AI-assisted technologies in the writing process

During the preparation of this work the authors used ChatGPT 4o to improve readability and language. After using this tool/service, the authors reviewed and edited the content as needed and take full responsibility for the content of the published article.

## CRediT authorship contribution statement

**Saul Martinez-Horta:** Writing – review & editing, Writing – original draft, Visualization, Validation, Supervision, Resources, Project administration, Methodology, Investigation, Funding acquisition, Formal analysis, Data curation, Conceptualization. **Angela Quevedo-García:** Writing – original draft, Project administration, Methodology, Investigation, Formal analysis. **Arnau Puig-Davi:** Writing – original draft, Supervision, Project administration, Formal analysis, Data curation. **Frederic Sampedro:** Writing – original draft, Visualization, Validation, Supervision, Resources, Methodology, Formal analysis, Data curation. **Javier Oltra-Cucarella:** Writing – review & editing, Writing – original draft, Visualization, Validation, Methodology, Formal analysis, Data curation. **Jesús Pérez-Pérez:** Resources, Project administration, Methodology, Investigation, Conceptualization. **Carla Franch-Martí:** Writing – original draft, Project administration, Methodology, Investigation, Formal analysis. **Gonzalo Olmedo-Saura:** Resources, Project administration, Methodology, Investigation, Conceptualization. **Elisa Rivas-Asensio:** Writing – original draft, Validation, Project administration, Methodology. **Anna Vazquez-Oliver:** Writing – original draft, Validation, Project administration, Methodology. **Laura Pérez-Carasol:** Writing – original draft, Validation, Project administration, Methodology. **Andrea Horta-Barba:** Writing – original draft, Validation, Project administration, Methodology. **Javier Pagonabarraga:** Writing – review & editing, Writing – original draft, Visualization, Validation, Supervision, Resources, Conceptualization. **Jaime Kulisevsky:** Writing – review & editing, Writing – original draft, Visualization, Validation, Supervision, Resources, Funding acquisition, Conceptualization.

## Declaration of competing interest

The authors declare that they have no known competing financial interests or personal relationships that could have appeared to influence the work reported in this paper.

## Data Availability

Data will be made available on request.
