# Supplementary material for: Consolidation deficits in episodic memory define distinct clinical and neurodegenerative profiles in Huntington’s disease
Source: Neuroimage Clin. 2025 Oct 28;48:103894. doi: 10.1016/j.nicl.2025.103894 (PMC12607079; doi:10.1016/j.nicl.2025.103894)

SUPPLEMENTARY DATA

Supplementary Figure 1: GMV differences between HC and HD

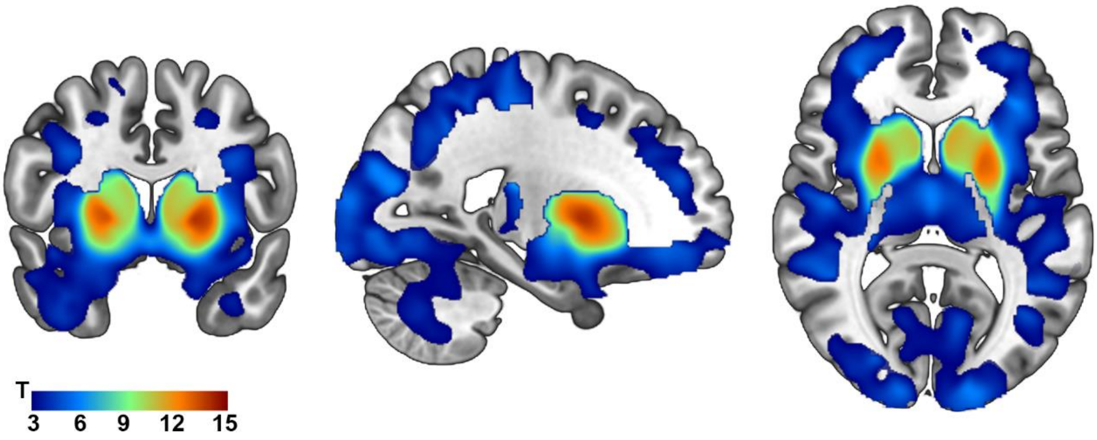

Supplementary Figure 2: Superposition of GMV differences between HC and HD-1 and between HC and HD-2

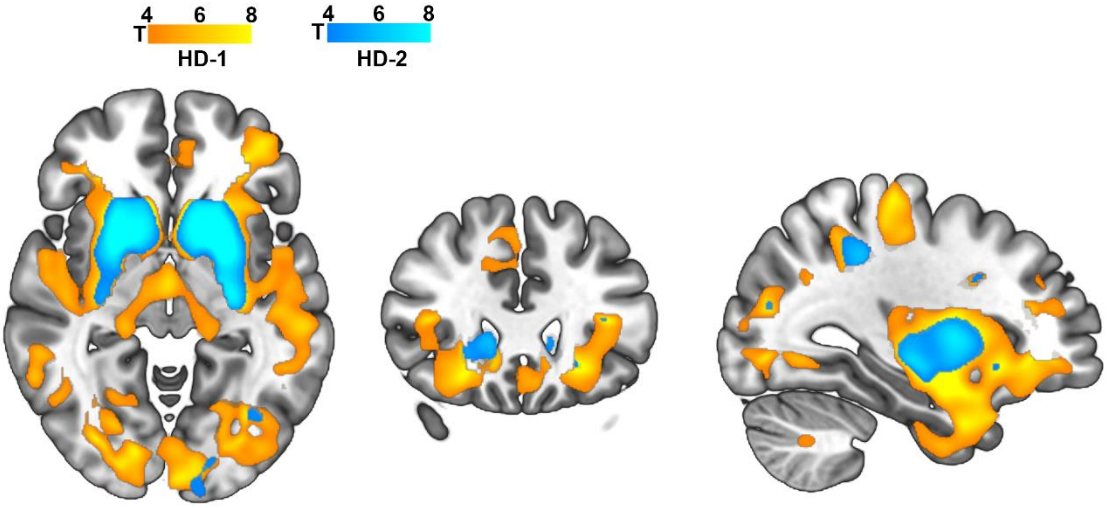

Supplement: Supplementary Data 1 [file mmc1.pdf]
